# Division of Labor Among Transgender and Gender Non-binary Parents: Association With Individual, Couple, and Children’s Behavioral Outcomes

**DOI:** 10.3389/fpsyg.2020.00015

**Published:** 2020-01-23

**Authors:** Samantha L. Tornello

**Affiliations:** Human Development and Family Studies, Pennsylvania State University, University Park, PA, United States

**Keywords:** transgender, division of labor, parents, relationship satisfaction, child behavior

## Abstract

The division of unpaid labor is an important aspect in understanding co-parenting dynamics, along with individual well-being, couple functioning, and family dynamics. This study explores the division of household and childcare unpaid labor, well-being, relationship functioning, and child behavioral outcomes in 163 transgender and gender non-binary (TGNB) parents. Research exploring the division of labor among cisgender heterosexual couples has found that cisgender women in heterosexual couples disproportionately conduct more of the household and childcare labor (e.g., Lachance-Grzela and Bouchard, 2010). In addition, among heterosexual (e.g., Lachance-Grzela and Bouchard, 2010) and same-sex couples (Tornello et al., 2015b), discrepancies in the division of unpaid labor has been associated with individual well-being, along with couple functioning. We know very little about the factors that predict how labor is divided, along with the impact these arrangements among of families headed by TGNB parents. In this study, TGNB parents reported dividing their household and childcare labor in an egalitarian fashion and wanted to divide their labor in that way. The gender of participants, gender design of the couple, educational attainment, and legal status of the couple’s relationship were not associated with the division of unpaid labor. In contrast, participants who reported making a lower proportion of the household income, worked less hours in paid employment, and were genetically related to their eldest child, reported completing significantly more childcare-related tasks, but not household labor. Using multiple regressions, participants’ genetic relatedness to their eldest child was the only significant predictor of performing greater unpaid childcare labor. Lastly, discrepancies in the household, but not childcare labor, predicted parental well-being and couple functioning. The division of labor among TGNB couples was unrelated to their child behavior outcomes. This study not only sheds light on the dynamics of TGNB-headed families, but also additional factors that influence the division of unpaid labor and how this division affects individuals within the family system.

## Introduction

Division of labor is typically defined as who performs the unpaid household (e.g., washing dishes, cleaning the house, doing laundry) and childcare (e.g., feeds the child, gets up with the child at night, and does homework with a child) tasks (Cowan and Cowan, 1992). How a couple divides their unpaid labor is essential for understanding couple and co-parenting dynamics (e.g., Cowan and Cowan, 1992; Coltrane, 2000; Lachance-Grzela and Bouchard, 2010). For cisgender heterosexual couples, household and childcare labor is typically specialized, with cisgender women doing disproportionally more of the unpaid labor, especially childcare, and men engaging in more paid labor outside the home (e.g., Coltrane, 2000; Lachance-Grzela and Bouchard, 2010). In contrast, for same-sex couples, the division of unpaid labor is reported to be much more egalitarian in nature (e.g., Goldberg et al., 2012; Farr and Patterson, 2013; Tornello et al., 2015a; Bauer, 2016; Brewster, 2017). Across all couples, it is not the actual division of unpaid labor that is associated with individual, couple, and child outcomes, but instead their satisfaction with how these tasks are performed (e.g., Coltrane, 2000; Lachance-Grzela and Bouchard, 2010; Tornello et al., 2015b). Extensive research has examined the division of labor in cisgender heterosexual couples, with a growing area of work exploring these dynamics among same-sex or sexual minority couples.

We know very little about family and relationship dynamics of couples where one or more members identifies as transgender and gender non-binary (TGNB), specifically their division of labor (for exceptions see Pfeffer, 2010; Kelly and Hauck, 2015). TGNB people are typically described as people whose gender differs from what is normatively expected of their sex assigned at birth (American Psychological Association, 2015). Approximately, between 0.3 and 0.6% of the United States population identifies as transgender, although this is likely a great underestimate (Flores et al., 2016; Meervijk and Sevelius, 2017) due a lack of questions on inclusion and standardization of gender identity and sex assigned at birth in research studies. Related, we do not know how many TGNB people are parents, but researchers estimate that between 18 and 50% of TGNB people are currently parents (Grant et al., 2011; Stotzer et al., 2014; James et al., 2016), with an increasing number of individuals who wish to become parents in the future (Light et al., 2017). The purpose of this study is to explore the division of household and childcare labor among TGNB parents, along with examining the factors that predict how these couples divide responsibilities and tasks, and the impact this division has on individual, couple, and child functioning.

As stated prior, for cisgender heterosexual couples, household and childcare labor is typically specialized based on gender, with cisgender women doing more of the unpaid labor, especially childcare labor, and men doing more of the paid labor outside the home (e.g., Coltrane, 2000; Lachance-Grzela and Bouchard, 2010). In contrast, sexual minority (or same-sex) couples report dividing their household and childcare labor in a more egalitarian way compared to their heterosexual peers (e.g., Goldberg et al., 2012; Farr and Patterson, 2013; Tornello et al., 2015b; Bauer, 2016; Brewster, 2017). We know that TGNB people conceptualize their sexual identity differently than cisgender identified people (Nagoshi et al., 2012; Galupo et al., 2016) and often see gender and gender role expectations as more fluid (Nagoshi et al., 2012). The ways in which cisgender heterosexual couples divide their unpaid labor are often shaped by gender constructions and roles (Erickson, 2005). Same-sex couples, on the other hand, seem to assign these tasks based on personal preferences and negotiation rather than gender (Kurdek, 2007). For TGNB people, is the division of unpaid labor based on gender role assumptions or couple gender design? To understand the factors that influence the division of unpaid labor among TGNB people, three major theories will be explored: relative resource theory (income and education), time-constraint theory (hours in paid employment), and life course theory (relationship status, length of relationship, and family design). Next, I will briefly describe each theory and review relevant literature in this area.

### Relative Resource Theory

According to relative resource theory, unpaid labor is divided based on the amount of resources, specifically the level of education and income each member of the couple brings to the relationship (Blood and Wolfe, 1960). In other words, the partner with higher educational attainment and individual income will perform less household and childcare labor. There is support for the relative resource theory among heterosexual couples: cisgender women typically report lower educational attainment and income compared to their partners, and in turn, perform more of the unpaid labor (e.g., Bianchi et al., 2000). Among sexual and gender minority couples, the research support for relative resource theory is mixed.

Among a sample of lesbian, gay, and heterosexual adoptive parents, partners reporting greater income disparities also reported greater incongruences in feminine-related household tasks (such as washing dishes or laundry as opposed to lawn or car maintenance) across all couple types (Goldberg et al., 2012). Related, in a study of 9 men and 40 women in same-sex relationships with school-aged children, partners who reported lower educational attainment, along with lower individual incomes, performed more of the school-related childcare tasks (Sutphin, 2013). In addition, Patterson et al. (2004) found that discrepancies in education, but not income, predicted who performed unpaid childcare labor among lesbian couples. In contrast, among childfree lesbian, gay, and heterosexual couples, Kurdek (1993) found support for the relative resource theory among heterosexual – but not gay and lesbian – couples. Related, for cisgender gay fathers, income and educational attainment did not predict the allocation of household or childcare labor (Tornello et al., 2015b). In all, relative resource theory seems to apply in same sex couples more often to childcare, but not household labor, although these results have not been consistent.

The majority of this work has focused on same-sex and/or sexual minority couples, with very little research exploring the experiences of TGNB couples. To date, only one study has explored the division of labor in TGNB couples as it relates to relative resources of the partners. In this qualitative study of 30 couples, income did play a role in the division of their unpaid labor, but it was not the strongest determinant (Kelly and Hauck, 2015). It is important to note that this study was qualitative in nature. It also consisted of a small sample, most were not parents and they did not examine the role of couple gender design (Kelly and Hauck, 2015). Findings regarding relative resource theory among sexual and gender minority couples is quite mixed, with very limited work exploring the experiences of TGNB couples. The principles of time-constraint theory have had more consistent support.

### Time-Constraint Theory

According to the time-constraint theory, the partner who works more hours in paid employment participates less in unpaid household and childcare labor (Presser, 1994; Silver and Goldscheider, 1994). A number of studies have found support for the time-constraint theory among heterosexual, gay, and lesbian cisgender couples (Patterson et al., 2004; Goldberg et al., 2012; Tornello et al., 2015b). In a study of gay fathers, when controlling for relative resources (e.g., income and education) of the couple as well as life course factors (e.g., length of relationship and family design), hours in paid employment was the only predictor of household division of labor. The results for childcare labor were much more complicated, but time in paid employment was still a large predictor in how much each partner contributed (Tornello et al., 2015b). In a study exploring the experiences of women in same-sex couples through the transition to parenthood, researchers found that genetic mothers did slightly more of the childcare, especially if they were working fewer hours in paid employment (Goldberg and Perry-Jenkins, 2007). There has been consistent support for time-constraint theory among all couple types, regardless of sexual or gender identity; therefore, it is hypothesized that the partner who works more hours outside the home in paid employment will perform less household and childcare tasks.

### Life Course Theory

Life course theory is the idea that experiences or decisions across the life course can impact or alter later development (Elder, 1998). As it relates to division of labor, life course theory has examined the ways in which relationship status, length of relationship, and family design can affect how couples designate their unpaid labor (e.g., Baxter et al., 2008; Grunow et al., 2012; Yavorsky et al., 2015; Bauer, 2016). Among cisgender heterosexual couples, the specialization of unpaid labor increases the longer the couple remains in a relationship, as well as when the couple becomes parents (e.g., Baxter et al., 2008; Grunow et al., 2012; Yavorsky et al., 2015). Findings were mixed for cisgender lesbian and gay couples (Kurdek, 2005; Tornello et al., 2015b; Bauer, 2016).

In a review, Kurdek (2005) proposed that same-sex couples who have been together longer would be more specialized in their division of unpaid labor. This was confirmed in an international study exploring the association between relationship length and division of labor, in which researchers found that the longer a couple was together, the more specialized the division of unpaid labor was (Bauer, 2016). This was less pronounced among men in same-sex couples (Bauer, 2016). In contrast, in a study discussed prior, relationship length among cisgender gay fathers was not predictive of how they divided their unpaid labor (Tornello et al., 2015b). These variations may be due to stronger associations between relationship length and parenthood in cisgender heterosexual couples. As a result, those in longer romantic relationships are also more likely to be parents. To date, we do not know if relationship length is associated with how TGNB couples divide their unpaid labor.

We do know that parenthood is associated with increases in specialization of division of labor (Bauer, 2016). It is important to note that for sexual and gender minority people, as compared to the majority of cisgender heterosexual couples, there are unique aspects of family design. For planned cisgender same-sex and TGNB headed families, many pathways to parenthood can result in one parent being genetically related to the child and one not (e.g., use of reproductive technologies where one partner or a surrogate carries the child), or neither (e.g., adoption or foster care). Genetic relatedness among same-sex planned families has not typically been associated with the couple’s division of household or childcare labor (Vanfraussen et al., 2003; Sutphin, 2013; Tornello et al., 2015a). Related, in a comparison of adoptive cisgender heterosexual, lesbian, and gay parents with no genetic ties to the focal child, heterosexual couples were more specialized compared to lesbian mothers and gay fathers (Goldberg et al., 2012). However, when examining genetic relatedness in the context of divorce or blended families, these findings are very different.

In exploring the division of unpaid labor among blended families, typically the genetic parent performs more of the childcare tasks compared to the non-genetic or stepparent (e.g., Moore, 2008; Tornello et al., 2015b). For example, in a study of women in same-sex blended families, the child’s genetic mother completed more of the childcare related tasks compared to the stepmother (Moore, 2008). In a similar study of cisgender gay fathers who became parents in the context of a prior heterosexual identity, the genetic father completed more childcare duties compared to the stepfather (Tornello et al., 2015b). Family design did not predict the division of unpaid household labor (Tornello et al., 2015b). Among heterosexual cisgender couples, stepparents consistently perform less of the unpaid labor (Ishii-Kuntz and Coltrane, 1992). Genetic relatedness to a child was not predictive of a couple’s division of labor, but being a genetic parent in a blended family was.

### Impact of Division of Labor on Individual Well-Being, Relationship Satisfaction, and Children’s Behavior

Who performs which household or childcare tasks does not often result in negative individual, couple, or family outcomes. Specifically, it is not the *type* of division – specialized vs. egalitarian, but the expectations of each member and their satisfaction with this division. If the couple decides on a more specialized division of labor because it more appropriately reflects their gender role ideation or partner expectations, this is not associated with negative outcomes. Research exploring the impact of discrepancies or disagreements over unpaid labor has focused on three major areas: individual well-being, couple functioning, and child adjustment (e.g., reviewed in Coltrane, 2000; Lachance-Grzela and Bouchard, 2010).

If each member of the couple has a strong desire for an equitable division of labor, but this is not occurring (Kalmijn and Monden, 2011), or if one partner is experiencing the majority of the stress related to these demands (Tao et al., 2010), this can result in a decreased sense of individual well-being. A similar association has been found among sexual minority or same-sex couples. As stated previously, same-sex couples report a more egalitarian division of labor compared to their heterosexual peers, but this alone does not result in negative well-being. In a study exploring the experiences of women in same-sex relationships during the transition to parenthood, Goldberg and Smith (2008) found that anxiety increased for both parents after the birth of the child, but that the causes were different for the genetic and non-genetic mothers. Specifically, the genetic mother who worked more hours in paid employment and was performing less of the childcare, expressed greater levels of anxiety (Goldberg and Smith, 2008). Again, well-being seems more likely to be affected by the *discrepancies* between ideal and actual division of unpaid labor. For example, in a study of 176 cisgender gay fathers that controlled for the actual division of unpaid labor, greater division of labor discrepancies predicted greater depressive symptoms and lower satisfaction with life (Tornello et al., 2015a). In all, greater discrepancies between actual and ideal division of unpaid labor have been linked to individual well-being.

Another aspect of family life that can be affected by the division of labor is relationship satisfaction or functioning. Greater perceived equalities or discrepancies in the division of unpaid labor have been associated with negative relationship outcomes among heterosexual couples (Coltrane, 2000; Saginak and Saginak, 2005; Mikula et al., 2012) and lesbian and gay couples (Kurdek, 2007; Sutphin, 2010; Tornello et al., 2015a). Among childfree same-sex couples, greater satisfaction with how the couple divides their unpaid labor was associated with greater relationship satisfaction (Sutphin, 2010). Related, gay cisgender surrogate fathers who reported lower discrepancies in unpaid labor seemed to enjoy greater relationship satisfaction (Tornello et al., 2015a). In sum, satisfaction with division of unpaid labor has an impact on relationship satisfaction and this has been found to be consistent across all couple types.

Prior work has also found associations between division of labor and children’s adjustment, often explained though the co-parent or couple functioning (e.g., Chan et al., 1998; Farr and Patterson, 2013). Research exploring the direct relationship between division of labor and children’s outcomes has had mixed findings (e.g., Patterson, 1995; Chan et al., 1998; Tornello et al., 2015b). Among heterosexual cisgender couples, mothers’ reports of less externalizing behaviors were associated with their partner’s reports of greater satisfaction with decision-making labor (Chan et al., 1998). No other associations between children’s behavioral outcomes and division of labor were found among the heterosexual couples (Chan et al., 1998). In two studies that explored the experiences of lesbian mothers based on genetic relatedness, greater satisfaction of the non-genetic mother regarding their division of childcare labor (Patterson, 1995) and family decision-making (Chan et al., 1998) was associated with better child adjustment. In a more recent study, discrepancies in division of labor among cisgender gay fathers were associated with individual well-being and relationship functioning, but were unrelated to their child behavioral outcomes (Tornello et al., 2015b). In contrast, in a study of adoptive cisgender heterosexual, lesbian, and gay adoptive parents, greater satisfaction with childcare was associated with less externalizing behaviors among the children (Farr and Patterson, 2013). For children’s outcomes, the ways in which a couple divides their labor and how satisfied they are with that labor, may not be directly associated with children’s outcomes, but rather, a reflection of larger relationship dynamics and couple functioning.

### Current Study

This study has three major aims: (1) Provide descriptive information regarding division of household and childcare labor among TGNB parents. Based on the prior findings that TGNB people hold more fluid and flexible ideas about gender identity, gender roles, and sexual orientation (Nagoshi et al., 2012; Galupo et al., 2016), TGNB parents will report dividing their household and childcare labor in an egalitarian fashion. Similarly, TGNB parents will have low discrepancies between their actual and ideal division of labor. In addition, as with sexual minority individuals (e.g., Goldberg et al., 2012; Farr and Patterson, 2013; Tornello et al., 2015b; Bauer, 2016; Brewster, 2017) and in contrast with cisgender heterosexual couples (e.g., Artis and Pavalko, 2003; Bauer, 2016), there will be no differences in the division of unpaid household and childcare labor across parental gender or couple gender design (same-gender vs. different gender couples). (2) Understand the factors that shape the division of household and childcare labor in TGBN couples. Three theoretical models will be used to predict division of labor. The relative resource theory will examine the role of income and education in division of household and childcare labor, with the hypothesis that income – but not education level – will predict household and childcare division of labor (e.g., Bianchi et al., 2000; Patterson et al., 2004; Goldberg et al., 2012; Sutphin, 2013; Kelly and Hauck, 2015). Next, consistent with time-constraint theory, the individual who works fewer hours in paid employment will complete more of the household and childcare unpaid labor (e.g., Patterson et al., 2004; Goldberg et al., 2012; Tornello et al., 2015b). The life course theory will be used to explore couple and family factors, such as length of relationship and family design (genetic vs. non-genetic parent). As has been found with research among same-sex couples (e.g., Vanfraussen et al., 2003; Moore, 2008; Sutphin, 2013; Tornello et al., 2015a, b), genetic parents will complete more childcare tasks, but not household labor, compared to non-genetic parents. (3) Explore the relationships between division of labor discrepancies and individual well-being, relationship satisfaction, and children’s behavioral outcomes. Household and childcare division of labor discrepancies, not current division of labor, will directly predict individual (Goldberg and Smith, 2008; Tornello et al., 2015b) and couple functioning (Kurdek, 2007; Sutphin, 2010; Tornello et al., 2015a), but not children’s outcomes (Tornello et al., 2015b).

## Materials and Methods

### Participants

The study sample consisted of 163 TGNB parents and their children. The original sample consisted of 311 TGNB parents and their children. Due to our interest in the division of labor around childcare, those who had children over the age of 18 (*n* = 79) or child age was missing (*n* = 8) were removed. Participants who were currently single (*n* = 38), who had multiple current partners (*n* = 20), or did not live together at least 50% of the time (*n* = 3) were removed. The final sample consisted of 163 transgender and non-binary parents.

Participants were on average 36 (*SD* = 6.37) years of age, and the majority self-identified as White/European American (88.3%). The socioeconomic class of participants varied greatly; however, the majority reported being a middle class household, having a Bachelor’s degree or higher (60.7%), and of those who were currently were employed, participants worked an average of 41 (*SD* = 9.88) h per week. Most participants identified their gender as transgender men (25.2%) and transgender women (30.7%). A minority of participants identified their gender as genderqueer (16.0%), non-binary (8.0%), gender non-conforming (6.1%), gender fluid (3.1%), multiple gender identities (3.7%), and additional identities (7.3%; e.g., agender, bigender, choose not to label, genderless, and two-spirited). Due to the small numbers of participants identifying with these genders identities, these were combined into a non-binary gender group (44.1%). The majority of participants self-identified their sexual identity as queer (28.2%), lesbian (16.6%), pansexual (16.0%), bisexual (12.9%), heterosexual (9.2%), choose not to label (3.1%), demisexual (3.1%), gay (2.5%), asexual (2.5%), and additional identities (6.0%; e.g., questioning, androsexual, attracted to women, female-bodied women, not sure, and multiple sexual identities). All participants had a current partner, and the majority were legally married (79.7%). Participants reported being with their partner for an average of 10 (*SD* = 5.75) years, and those with a legally recognized relationship had been together for an average of 7 (*SD* = 5.30) years.

Participants’ partners were 37 (*SD* = 7.06) years old on average, self-identified as White/European American (85.3%), the majority had a Bachelor’s degree or higher (57.7%), and those who were employed worked an average of 39 (*SD* = 13.01) h per week. Participants identified their partners’ gender identities as predominantly cisgender women (65.6%), with others identifying as cisgender men (12.3%), transgender women (5.8%), transgender men (4.5%), gender non-conforming (4.0%), genderqueer (3.3%), multiple gender identities (1.3%), and additional gender identities (2.8%; e.g., gender fluid, non-binary, two-spirited, or choose not to label). As with participants, if the partner identified their gender identity as non-binary (e.g., gender non-conforming, genderqueer, non-binary, or reported multiple gender identities) they were grouped in the non-binary gender group (11.4%). Participants reported their partner’s sexual identity as heterosexual (31.6%), queer (19.1%), bisexual (13.2%), pansexual (13.2%), lesbian (11.2%), choose not to label (3.9%), gay (2.6%) and some other sexual identity (5.5%; e.g., asexual, demisexual, polysexual, questioning, or unknown).

Participants reported having an average of two children per family (*SD* = 0.97). The eldest children of participants joined their families in many different ways. Most children were conceived through genetic means (96.3%), with a few joining the family through adoption (2.5%) and foster care (1.2%). In the subset of participants who had children join their family through genetic means, over half the participants and their current/former partners were genetically related to the focal child (53.8%), 30.1% of participants were not genetically related but their current/former partner was genetically related, and 16.1% of participants were genetic parents without any genetic co-parent. On average, eldest children were approximately 8 (*SD* = 5.47) years of age, most participants identified their children’s race/ethnicity as White/European American (81.6%), and about half were assigned female at birth (49.1%). All demographic information is on Table 1.

**TABLE 1:** Demographic information of Transgender and Non-binary parents, partners, and eldest child.

	**Participant *n* = 163**	**Partner *n* = 163**	**Child *n* = 163**
**Variable**	***M (SD)***	***M (SD)***	***M (SD)***
Age	36.15 (6.37)	37.72 (7.06)	7.59 (5.47)
Individual income (thousands)	49,750.96 (52,375.32)	–	–
Household income (thousands)	86,427.12 (79,170.22)	–	–
Hours per week in paid employment	33.58 (17.74)	29.78 (19.36)	–
Length of relationship (years)	10.05 (5.75)	–	–
Number of children	1.71 (0.99)	–	–
Sex assigned at birth (% female)	62.6	78.1	49.1
Gender (%)			
Transgender woman	25.8	3.7	0.0
Transgender man	21.5	2.5	0.0
Cisgender woman	4.9	62.6	38.7
Cisgender man	3.7	12.9	35.0
Genderqueer	16.0	3.1	0.0
Gender non-conforming	6.1	3.7	1.2
Gender fluid	3.1	0.6	1.2
Non-binary	8.0	0.6	0.0
Multiple identities	3.7	1.2	0.0
Choose not to label/unknown	0.6	0.6	18.4
Additional identities^a^	6.7	0.6	5.5
Sexual orientation (%)			
Queer	28.2	17.8	–
Heterosexual	9.2	29.4	–
Lesbian	16.6	10.4	–
Gay	2.5	2.5	–
Bisexual	12.9	12.3	–
Pansexual	16.0	12.3	–
Asexual	2.5	0.6	–
Demisexual	3.1	0.6	–
Questioning	1.2	1.2	–
Choose not to label	3.1	3.7	–
Not sure/unknown	0.6	0.6	–
Additional identities^b^	4.2	1.8	–
Race/ethnicity			
White/Caucasian	87.7	78.5	81.6
Hispanic/Latino(a)	3.7	4.9	4.3
Black African American	0.0	1.2	0.6
Asian Indian	1.2	1.2	0.6
Biracial/Multiracial	6.7	1.8	9.2
Additional race/ethnicities^c^	0.0	4.2	1.2
Relationship status			
Committed relationship	14.1	–	–
Married legally recognized	79.1	–	–
Engaged	6.1	–	–
Polyamorous	0.6	–	–
Education			
Less than high school	1.2	1.8	–
High school/GED	23.9	25.2	–
Vocational/Trade school	3.1	3.1	–
Associates degree/2 years	11.0	9.2	–
Bachelor’s degree/4 years	24.5	23.3	–
Graduate degree	36.2	30.1	–

*Not all numbers will total to 100 due to rounding. ^*a*^Additional gender identities include: agender, bigender, genderless, trans feminine, and androgynous. ^*b*^Additional sexual identities include: omnisexual, transitioning sexual orientation, heteroflexible, polysexual, female bodied women, dike, androsexual, and attracted to women. ^*c*^Additional race/ethnicities include: American Indian/Alaskan Native, Chinese, Filipino, Native Hawaiian/Pacific Islander, and Creole.*

### Procedure

Participants were recruited through a large international study of gender-diverse parents and their children. Study advertisements were listed on social media and networking websites for transgender and gender non-conforming/non-binary parents. The inclusion criteria for the study was that the individual had to identify their gender as non-cisgender, be a parent of at least one child, and be over the age of 18. Participants saw advertisements that included the inclusion criteria on family and parenting TGBN websites, and if they were interested in participating they contacted the PI (author) of the study or completed an online information form. If eligible to participate, they received an email with a personalized study link and password for them and their partner (if applicable). When clicking on the link, participants first read the consent form, agreed to participate, and then completed a series of surveys. The study proposal, consent, and surveys were approved by the IRB at Pennsylvania State University.

### Measures

#### Demographics

Participants completed a series of demographic questions about themselves and their partners, such as age, gender, sex assigned at birth, sexual orientation, race/ethnicity, individual and household income, hours of paid employment per week, educational attainment, relationship status, and religious affiliation. Participants were asked a series of question about their eldest child such as age, gender, sex assigned at birth, race/ethnicity, and how the child joined the family.

#### Pathways to Parenthood

Participants completed a series of questions about how their eldest child joined the family. Participants were first asked “Which of the following best describes how this child came into your family?” The question included the following response options: “I and/or my partner (or former partner) is biologically related to the child,” “Through adoption (no direct biological relationship with the child; this option includes foster care to adoption situations),” “Through the foster care system (either or both is the legal foster parent),” or “Self-describe (please specify).” If participants choose the option of genetically related, they were asked “Which best describes your current situation?” with the options regarding who is genetically related to the child (participant, partner, another individual) and the means of conception.

#### Division of Household and Childcare Labor

Participants completed the Who Does What (WDW) Scale (Cowan and Cowan, 1992, 1995), which measures a couple’s division of labor. Two types of division of labor were assessed: household division of labor, such as preparing meals, laundry, and cleaning the home (13 items), and childcare division of labor based on the age of the child (six versions; 12–20 items depending on the age of the child), such as dressing, homework, and organizing playdates. For each item, participants rated on a 9-point Likert scale (1 = *partner does it all* to 9 = *I do it all*) based on who completes the tasks (actual) and how the participant would like it to be (ideal).

Six different scores were calculated: (a) actual household labor was calculated by taking the average of actual household items; (b) actual childcare labor was calculated by taking the average of actual childcare items; (c) ideal household labor was calculated by taking the average of ideal household items; (d) ideal childcare labor was calculated by taking the average of ideal childcare items; (e) discrepancy scores in household division of labor were calculated by taking the average of the absolute difference of the actual and ideal household responses; and (f) discrepancy scores in childcare division of labor were calculated by taking the average of the absolute difference of the actual and ideal childcare responses. A score closer to five on the actual household or childcare division of labor reflected an egalitarian division of labor. A score closest to five on the ideal household or childcare division of labor reflects a desire to have an egalitarian division of labor. On the discrepancy division of household and childcare labor scales, values closer to zero reflected greater similarity between how the labor was being divided and how the individual would ideally like it to be divided. All scales had moderate to high reliability (alphas = 0.62 through 0.92).

#### Individual Well-Being

Participant well-being was measured using two different scales. First, the Center for Epidemiological Studies-Depression Inventory (CES-D; Radloff, 1977), a 20-item self-report survey that measures the frequency of symptoms associated with depression, was administered. Participants were asked how often in the past week they felt lonely, talked less than usual, or had crying spells. Participants responded to each item using a 4-point Likert scale [0 = *Rarely or none of the time (less than 1 day)*, 1 = *Some or a little of the time (1–2 days)*, 2 = *Occasionally or a moderate amount of the time (3–4 days)*, and 3 = *Most or all of the time (5–7 days)*]. A total score was calculated by summing all item responses for a total score that ranged from 0 to 60, with scores >16 (Radloff, 1977) reflecting clinical levels of depressive symptoms. This scale had good reliability (alpha = 0.90).

The second measure used was the Satisfaction with Life Scale (Diener et al., 1985), a 5-item self-report survey that measures an individual’s current level of contentment with their life. Example items include, “The conditions of my life are excellent,” “So far I have gotten the important things I want in life,” and “In most ways my life is close to my ideal.” Participants responded to survey items using a 7-point Likert scale from 1 = *Strongly disagree* to 7 = *Strongly agree*. A total score was calculated by summing all responses that could range from 5 to 35. This scale had good reliability with an alpha of 0.88.

#### Relationship Adjustment

The Dyadic Adjustment Scale (DAS; Spanier, 1976) is a 32-item survey used to measure the participants’ relationship adjustment with their current romantic partner. Items addressed different aspects of a romantic relationship such as, “In general, how often do you think that things between you and your partner are going well?” or “How often do you or your mate leave the house after a fight?” Item response scales varied, with some items having 6-point Likert scales in which 0 = *never* and 5 = *more often* or 0 = *always disagree* and 5 = *always agree*, or a 2-point scale, such that 0 = *yes* and 1 = *no*. An overall relationship adjustment score was calculated by summing all item responses together to create a total score, which could range from 0 to 151, with higher scores reflecting greater relationship adjustment. Previous research has found that the average score in a heterosexual married sample was 114.8 ± 17.8 (Spanier, 1976). This sample was within normal range with a total score of 112.30 ± 13.64. This scale had good reliability with this sample, with an alpha of 0.90.

#### Child Behavior

The Child Behavior Checklist (CBCL; Achenbach and Rescorla, 2000, 2001) measured children’s behavioral and emotional development. Two versions of the CBCL were used depending on the child’s age, with the preschool version (100 problem behavior items) being used among children ages 1 1/2 to 5 years of age, and the school age version (118 problem behavior items) for children 6 to 18 years of age (Achenbach and Rescorla, 2000, 2001). Example items for the preschool version include behaviors such as, “*cries a lot*,” *“unusually loud,”* “*disobedient at home*,” and “*argues a lot*.” Items for the preschool version included behaviors such as, “*acts too young for age*,” “*defiant*,” “*easily frustrated*,” *“worries,”* and *“sulks a lot*.” Participants responded to each item using the Likert scale of in which 0 = *Not true*, 1 = *Somewhat or sometimes true*, or 2 = *Very true or often true*. All responses were totaled for a final behaviors score. Scores were then standardized based on the child’s age and sex assigned at birth using the Achenbach System of Empirically Based Assessment (ASEBA^®^ Web^TM^) online scoring system (Achenbach, 2010). Both the CBCL preschool version (alpha = 0.95) and the school-age version (alpha = 0.94) had good reliability.

## Results

Findings are presented according to the aims of the study. First, descriptions of the division of household and childcare unpaid labor and satisfaction with that division, along with conducting a series of one-way ANOVAs comparing the actual and ideal division of household and childcare labor based on individual and couple gender design will be explored. Second, multiple regression models will explore the predictors of household and childcare division of labor, while controlling for participant age, number of children, and age of eldest child. The three theoretical frameworks that will be tested are the relative resource theory (income and educational attainment), time-constraint theory (hours in paid employment), and life course theory (length of romantic relationship and family design). Finally, multiple regression models will explore if discrepancies in actual and ideal division of household and childcare labor-predict parental well-being, relationship satisfaction, and children’s adjustment.

### Division of Household and Childcare Labor

On average, participants reported having (*M* = 5.48, *SD* = 0.98) and wanting (*M* = 5.10, *SD* = 0.57) an egalitarian division of household unpaid labor. Similarly, participants reported having (*M* = 5.24, *SD* = 1.24) and wanting (*M* = 5.03, *SD* = 0.84) an egalitarian division of childcare labor. When examining the discrepancies in the division of household and childcare labor, participants reported being satisfied, *M* = 0.72, *SD* = 0.61, *M* = 1.19, *SD* = 0.75, respectively.

Division of labor was then examined by participant gender by comparing three groups: Transgender men (25.2%), transgender women (30.7%), and non-binary (44.1%). Current and desired division of household labor did not differ by parent gender, *F*_(__2_,_141__)_ = 0.55, *p* = 0.58, *F*_(__2_,_135__)_ = 0.17, *p* = 0.85; see Table 2. Similarly, there were no differences in current and desired division of childcare labor, *F*_(__2_,_126__)_ 1.06, *p* = 0.35, *F*_(__2_,_122__)_0.13, *p* = 0.88; see Table 2. Discrepancies in division of both household and childcare labor did not differ by parent gender, *F*_(__2_,_122__)_ = 0.23, *p* = 0.80, *F*_(__2_,_135__)_ = 0.32, *p* = 0.73; see Table 2. To examine parental gender by couple design, couples were split into two groups: (1) those with the same gender identities (e.g., both members identified as men, women, or GNB) or (2) different gender identities (e.g., one member identifies as a man and one as a woman). There was no difference in current or ideal division of household or childcare based on partner gender design (*p* < 0.14).

**TABLE 2:** Division of household and childcare actual and ideal labor among TGNB parents by gender identity.

**Variable**	**Male *M (SD)***	**Female *M (SD)***	**Non-binary *M (SD)***	***F***
Household actual division of labor^a^	5.59 (0.93)	5.36 (1.07)	5.50 (1.07)	n.s.
Household want division of labor^a^	5.09 (0.61)	5.14 (0.56)	5.07 (0.56)	n.s.
Childcare actual division of labor^a^	5.14 (1.17)	5.06 (1.44)	5.41 (1.13)	n.s.
Childcare want division of labor^a^	5.01 (0.99)	5.10 (0.92)	5.01 (0.69)	n.s.
Ideal household division of labor^b^	1.10 (0.65)	1.20 (0.84)	1.23 (0.74)	n.s.
Ideal childcare division of labor^b^	0.66 (0.51)	0.72 (0.68)	0.60 (0.63)	n.s.

*^*a*^1 = *partner does it all* to 9 = *I do it all*. ^*b*^Higher values indicate greater division of labor discrepancies.*

### Predictors of Division of Labor

To understand the division of household and childcare labor of TGNB parents, three different theories – relative resource theory (income and education), time-constraint theory (hours in paid employment), and life course theory (relationship status, length of relationship, and family design) – were tested using a regression model, while controlling for participant age, number of children, and age of eldest child.

The first model predicting current household division of labor was not significant, *F*_(__9_,_101__)_ = 1.33, *p* = 0.08, with no controls or theoretical variables predicting current household division of labor. In contrast, participants who reported wanting to contribute more to the household division of labor were older, made a higher percentage of household income, and worked fewer hours in paid employment, *F*_(__9_,_96__)_ = 1.98, *p* = 0.049. The next two models examined the predictors of current and ideal childcare division of labor. Participants who reported currently performing more childcare tasks were in newer relationships, worked fewer hours per week in paid employment, and were the genetic parent to the focal child, *F*_(__9_,_91__)_ = 5.30, *p* < 0.001. For the ideal childcare division of labor, being the genetic parent to the focal child was the only significant predictor of desired division of childcare labor, *F*_(__9_,_87__)_ = 2.73, *p* = 0.008.

### Impact of Division of Labor on Individual, Couple, and Child Outcomes

Using a series of regression analyses, we explored the relationship between household and childcare division of labor discrepancies and individual, couple, and child outcomes. All models controlled for participant age, number of children, child’s age and actual division of labor, along with marital status, relationship length, parental genetic relatedness, relative education, proportion of income, hours in paid employment, and current division of labor (see Tables 3, 4).

**TABLE 3:** Division of labor household discrepancies predicting individual well-being, couple functioning, and child behavior among TGNB parents.

	**Depressive symptoms^a^**			**Satisfaction with life^b^**			**Relationship quality^c^**			**Child behavior^d^**		
**Variable**	***B***	***SE B***	**β**	***B***	***SE B***	**β**	***B***	***SE B***	**β**	***B***	***SE B***	**β**
Participant age	0.00	0.00	−0.24*	0.00	0.00	–0.09	0.00	0.00	–0.12	0.00	0.00	−0.36**
Number of children	–2.98	1.33	−0.23*	0.92	0.86	0.11	–1.18	1.70	–0.07	–3.03	2.33	–0.14
Eldest child age	0.44	0.26	0.19	–0.22	0.16	–0.15	0.55	0.32	0.20	1.33	0.44	0.35**
Married ^e^	0.38	3.57	0.01	1.12	2.33	0.05	–0.41	4.46	–0.01	–3.13	6.35	–0.05
Length of current relationship	–0.01	0.20	–0.01	0.19	0.13	0.15	–0.50	0.26	–0.20	0.46	0.36	0.14
Genetic relatedness to child^ f^	3.97	2.25	0.16	–2.14	1.46	–0.14	–1.70	2.84	–0.06	0.44	3.94	0.01
Relative education^ g^	0.28	0.56	0.05	0.63	0.36	0.17	1.01	0.71	0.15	–0.66	0.97	–0.07
Relative income^ g^	–0.11	0.07	–0.30	0.03	0.04	0.12	0.00	0.08	0.01	–0.06	0.12	–0.10
Relative hours worked in paid employment^ g^	0.10	0.08	0.23	–0.04	0.05	–0.14	–0.10	0.10	–0.17	0.18	0.14	0.24
Actual division of household labor^ h^	–1.90	1.27	–0.15	0.31	0.83	0.04	–1.48	1.62	–0.10	0.38	2.27	0.02
Division of household labor discrepancy^ i^	5.51	1.50	0.36***	–3.81	0.98	−0.39***	–6.05	2.04	−0.30**	1.38	2.67	0.05
*R*^2^	0.19			0.12			0.16			0.06		
*F*	3.04**			2.28*			2.64**			1.62		

^*a*^Higher scores indicate greater depressive symptoms. ^*b*^Higher scores indicate greater satisfaction with life. ^*c*^Higher scores indicate positive relationship quality. ^*d*^Higher scores indicate greater behavioral problems. ^*e*^1 = *Married*, 0 = *Not married*. ^*f*^1 = *Genetically related to focal child*, 0 = *Not genetically related to focal child*. ^*g*^Higher values indicate greater individual income, education, and hours worked in paid employment relative to their partner. ^*h*^Higher scores indicate greater participant participation in household division of labor. ^*i*^Higher values indicate greater household division of labor discrepancies. *B* = unstandardized beta. *SE B* = standard error for the unstandardized beta. β = standardized beta. *p < 0.05. **p < 0.01. ***p < 0.001.

**TABLE 4:** Division of labor childcare discrepancies predicting individual well-being, couple functioning, and child behavior among TGNB parents.

	**Depressive symptoms^ a^**			**Satisfaction with life^ b^**			**Relationship quality^ c^**			**Child behavior^ d^**		
**Variable**	***B***	***SE B***	**β**	***B***	***SE B***	**β**	***B***	***SE B***	**β**	***B***	***SE B***	**β**
Participant age	0.00	0.00	–0.18	0.00	0.00	–0.14	0.00	0.00	–0.17	0.00	0.00	−0.25*
Number of children	–3.84	1.43	−0.29**	1.63	0.93	0.19	–1.07	1.85	–0.07	–1.64	2.30	–0.08
Eldest child age	0.58	0.27	0.25*	–0.29	0.17	–0.20	0.38	0.34	0.13	1.58	0.43	0.42***
Married^ e^	2.40	3.78	0.07	–0.31	2.48	–0.01	–1.83	4.76	–0.04	–1.14	6.09	–0.02
Length of current relationship	–0.21	0.24	–0.10	0.23	0.16	0.18	–0.36	0.31	–0.14	0.06	0.39	0.02
Genetic relatedness to child ^f^	4.46	2.59	0.19	–1.89	1.71	–0.13	0.13	3.31	0.00	1.86	4.18	0.05
Relative education ^g^	0.64	0.62	0.11	0.36	0.41	0.10	1.09	0.79	0.16	–0.40	1.01	–0.04
Relative income ^g^	–0.18	0.07	−0.51*	0.06	0.05	0.26	0.01	0.09	0.01	–0.07	0.12	–0.13
Relative hours worked in paid employment ^g^	0.14	0.08	0.33	–0.06	0.06	–0.21	–0.13	0.11	–0.24	0.20	0.14	0.28
Actual division of childcare labor ^h^	–1.99	1.29	–0.20	0.04	0.86	0.01	0.13	1.66	0.01	1.53	2.11	0.10
Division of childcare labor discrepancy ^i^	4.58	2.26	0.23*	–1.63	1.50	–0.13	–6.30	2.93	−0.26*	2.95	3.69	0.09
*R*^2^	0.13			0.01			0.08			0.09		
*F*	2.28*			1.10			1.70^†^			1.91^†^		

^*a*^Higher scores indicate greater depressive symptoms. ^*b*^Higher scores indicate greater satisfaction with life. ^*c*^Higher scores indicate positive relationship quality. ^*d*^Higher scores indicate greater behavioral problems. ^*e*^1 = *Married*, 0 = *Not married*. ^*f*^1 = *Genetically related to focal child*, 0 = *Not genetically related to focal child*. ^*g*^Higher values indicate greater individual income, education, and hours worked in paid employment relative to their partner. ^*h*^Higher scores indicate greater participant participation in childcare division of labor. ^*i*^Higher values indicate greater childcare division of labor discrepancies. *B* = unstandardized beta. *SE B* = standard error for the unstandardized beta. β = standardized beta. ^†^*p* < 0.10. *p < 0.05. **p < 0.01. ***p < 0.001.

The first pair of models explored the predictors of participants’ depressive symptoms. Division of household labor discrepancies was the only factor that predicted participant depressive symptoms, *F*_(__11_,_88__)_ = 3.04, *p* = 0.002, adjusted *R*^2^ = 0.19. Reporting greater division of childcare labor discrepancies was predictive of depressive symptoms in the participant, as was having more children, having children who were older, and reporting a lower income, *F*_(__11_,_82__)_ = 2.28, *p* = 0.017, adjusted *R*^2^ = 0.13. Division of household labor discrepancies was the only factor that predicted participant satisfaction with life, *F*_(__11_,_91__)_ = 2.28, *p* = 0.017, adjusted *R*^2^ = 0.23. In contrast, division of childcare labor discrepancies were not predictive of the participants’ satisfaction with life, *F*_(__11_,_84__)_ = 1.10, *p* = 0.373. The next two models examined the predictors of relationship satisfaction. Participants who reported greater discrepancies in household division of labor reported lower relationship satisfaction, *F*_(__11_,_85__)_ = 2.64, *p* = 0.006, but relationship satisfaction was not associated with discrepancies in childcare division of labor, *F*_(__11_,_79__)_ = 1.70, *p* = 0.088. Lastly, neither household, *F*_(__11_,_94__)_ = 1.62, *p* = 0.106, nor childcare, *F*_(__11_,_85__)_ = 1.91, *p* = 0.05, discrepancies in division of labor were predictive of children’s behavioral problems.

## Discussion

In this study exploring the division of household and childcare labor of TGNB parents, there were a number of interesting findings. TGNB parents reported dividing their household and childcare labor in egalitarian ways, with this division being uninfluenced by gender or couples design. In exploring three theories used to predict division of labor, there was clear support for the time-constraint theory and the life course theory, with little support for the relative resource theory. Actual division of labor were not predictive of individual, couple, or child outcomes, but discrepancies in the ideal and actual division of this labor, specifically household labor, did predict individual well-being and couple functioning. Division of labor discrepancies were not predictive of child behavioral outcomes.

Similar to cisgender sexual minority couples (Goldberg et al., 2012; Farr and Patterson, 2013; Tornello et al., 2015b; Bauer, 2016; Brewster, 2017) and, in contrast, to cisgender heterosexual couples (Artis and Pavalko, 2003; Bauer, 2016), TGBN couples reported wanting – and actually having – an egalitarian division of household and childcare labor. In addition, discrepancies between how these couples actually and ideally wanted to divide this labor were relatively minimal. As hypothesized, and in contrast to cisgender heterosexual couples (Erickson, 2005), participants’ gender and the gender design of the couple did not play a role in how unpaid labor was divided. One explanation for these findings is that TGBN people conceptualize gender, gender role expectations, and sexual identity in a more fluid and dynamic fashion (Nagoshi et al., 2012; Galupo et al., 2016). This greater gender and sexual identity flexibility could lead TGNB couples to negotiate and decide the division of unpaid labor based on personal preferences, similar to cisgender same-sex couples (Kurdek, 2007), and in contrast with cisgender heterosexual couples. With cisgender heterosexual couples’ division of unpaid labor typically being shaped by gender role expectations or assumptions (Erickson, 2005). Although TGNB parents reported dividing their labor in an egalitarian fashion and wanting it to be that way, this division was not associated with couple gender. Additional factors also that predicted actual and ideal division of unpaid labor.

When examining the factors associated with how a couple divides their unpaid labor, there was limited support for relative resource theory but moderate support for the time-constraint and the life course theories. For these couples, relative income and hours in paid employment predicted ideal – but not actual – division of household labor. Specifically, if the participant reported a higher income and working more hours in paid employment relative to their partner, they reported wanting to perform less household labor. In the one qualitative study of TGNB couples, Kelly and Hauck (2015) found an association between individual income and household division of labor, although only one of the 10 TGNB couples were actually parents and this study did not control for other factors such as time in paid employment or genetic relatedness. As expected, these findings are in contrast with the research among cisgender heterosexual couples (Bianchi et al., 2000), but the findings do support some of the research on same-sex couples. Among cisgender gay men with children under the age of 18 and childfree lesbian and gay couples (Kurdek, 1993; Tornello et al., 2015b), income and education were not associated with household division of labor. Although among the cisgender gay fathers, when controlling for income and education, time in paid employment was associated with household division of labor (Tornello et al., 2015b). We could hypothesize that income and educational attainment are important at specific periods of time, and that having children may change the impact of these factors on the division of household labor among these couples.

For childcare labor, these findings were a bit more complex. As predicted, the genetically related parent who worked fewer hours in paid employment reported performing more of the childcare labor. Related, only genetic relatedness was associated with the ideal childcare labor, with genetic TGNB parents wanting to do perform more of the childcare labor. Prior research with same-sex couples has consistently found that the partner who works more in paid employment performs less of the childcare labor (Patterson et al., 2004; Goldberg et al., 2012; Tornello et al., 2015b), but the findings regarding genetic relatedness were more mixed (e.g., Vanfraussen et al., 2003; Goldberg and Perry-Jenkins, 2007; Moore, 2008; Sutphin, 2013; Tornello et al., 2015a, b). Among cisgender adoptive parents, in which genetic relatedness is not a factor, heterosexual couples reported being more specialized compared to their lesbian and gay peers (Goldberg et al., 2012). Genetic relatedness and childcare division of labor may be explained by family context, such as parenting in blended or stepfamilies (e.g., Ishii-Kuntz and Coltrane, 1992; Moore, 2008; Tornello et al., 2015b). Although these couples reported a generally egalitarian division of childcare labor, genetic relatedness and hours in paid employment both play a role in how childcare responsibilities were divided.

As hypothesized, regardless of how TGNB parents divide their unpaid labor, greater discrepancies between each partner’s actual and ideal division of unpaid labor, were associated with poorer individual well-being and couple relationship quality, but not child outcomes. Specifically, TGNB parents that reported greater discrepancies between their actual and ideal household and childcare labor reported greater depressive symptoms. In addition, couples with greater discrepancies in their household division of labor, but not childcare, reported greater overall life satisfaction. These findings replicate previous research, with more significant discrepancies between how unpaid labor is divided and the individual expectations of this division, resulting in more negative individual well-being (e.g., Coltrane, 2000; Goldberg and Smith, 2008; Lachance-Grzela and Bouchard, 2010; Tornello et al., 2015b). Prior research has also found that these inequalities impact relationship functioning, with greater discrepancies predicting poorer relationship functioning and satisfaction among cisgender heterosexual (e.g., Saginak and Saginak, 2005; Mikula et al., 2012) and same-sex couples (Kurdek, 2007; Sutphin, 2010; Tornello et al., 2015a). Some prior work with same-sex couples has found an association between child’s outcomes and satisfaction with childcare division of unpaid labor (Patterson, 1995; Chan et al., 1998), studies exploring household and childcare discrepancies directly, like this study, have not found this relationship (Tornello et al., 2015b). It is possible that satisfaction with division of labor, along with the co-parenting relationship mediated the association between children behavioral outcomes and unpaid labor (Chan et al., 1998; Farr and Patterson, 2013), which was unexplored in the current study. In sum, for all couples regardless of gender identity, if each partner believes their unpaid labor is divided the way they would like it to be, both the individual and couple enjoy greater functioning. This was not, however, directly related to children’s adjustment.

This study has a number of strengths and limitations. Research examining TGNB couples, especially parents, is quite scant (exceptions see Kelly and Hauck, 2015). This study was the first to explore both household and childcare division of labor qualitatively among a relatively large sample of TGNB parents. This sample of TGNB parents was heterogeneous in a number of ways, such as in parent gender, child age, and family design, but even with this diversity, some of these factors could not be examined in detail. For example, comparisons across gender identity were possible for some groups, but finer analyses of those who identified on the non-binary spectrum were not possible in this study. Future research should examine the experiences of people who identify as these less represented or with multiple gender identities. Related, although an examination of same-gender and different-gender couples were possible in this study, and exploration by sexual identity or orientation was not due to small sample sizes. Future work should examine the relationship between genders, along with sexual orientation, to provide a more complex examination of these family dynamics. Another limitation is that all participants identified their genetic relatedness to the focal child, but we do not know details regarding how that child joined the family, such as in the context of a current or former relationship. Future research should focus on the variations of family and couple dynamics based on family context. Related, it is important to note that this study was cross-sectional, along with being on-line and survey-based. Some researchers have discussed the shortcomings of self-report measures of division of labor (Carrington, 1999) since this division of unpaid labor could be shifting daily, weekly, or monthly, which would not be captured by this type of methodology. Future research should examine these constructs using multiple methods of data collection, including collecting data in real time with the use of daily diary methodology and observational techniques.

In all, this study provides insight into the couple and family dynamics of TGNB parents. TGNB parents report dividing their unpaid household and childcare labor in a generally egalitarian fashion, and report wanting it to be divided in that way. Parent gender, along with the sex and gender design of the couple, were not associated with how the couple’s unpaid labor was divided. Relative resources of each partner were not predictive of how the couple divided their unpaid labor, although time spent in paid employment and genetic relatedness was associated with the division of childcare labor. Regardless of how the couple divided their labor, fewer discrepancies between how the unpaid labor is being divided and how they would like it to be was predictive of better individual well-being and relationship quality, but unrelated to their children’s adjustment.

## Data Availability Statement

The datasets analyzed in this article are not publicly available. Requests to access the datasets should be directed to Samantha L. Tornello at SLT35@psu.edu.

## Ethics Statement

The studies involving human participants were reviewed and approved by Pennsylvania State University, Ethics Board (IRB). The patients/participants provided their written informed consent to participate in this study.

## Author Contributions

ST independently designed the study, research questions, data collection, and data analysis.

## Conflict of Interest

The authors declare that the research was conducted in the absence of any commercial or financial relationships that could be construed as a potential conflict of interest.
